# Clinical Society

**Published:** 1897-04-03

**Authors:** 


### CLINICAL SOCIETY.

Dr. W. Pasteur gave an account of an epidemic of infantile paralysis among children of the same family. Seven children were affected within the space of ten days. In all there were febrile manifestations with marked headache, which in three cases were followed by paralysis and in two by tremors, the latter being of short duration. One case was a typical one of infantile paralysis, another was of hemiplegic type and apparently of cerebral origin, while a third presented, in addition to a paralysis of spinal type, marked spasticity at the outset. It was, he thought, a matter of very great interest to consider what was the nature of these cases. It seemed clear that whatever their origin they were all casually connected with the primary fever, and that they were the result of the operation of some specific infective process. This certainly was not one of the eruptive fevers, nor was it diphtheria, and there was no influenza in the district, besides which the health of the parents had remained good, and the children themselves had shown no symptoms of catarrh. In view, then, of the fact that, whatever these cases were, they were the result of the same infective process, while one of them presented the usual features of infantile paralysis, it seemed to Dr. Pasteur that the morbid change was probably of the same nature as that which occurred in anterior poliomyelitis, but occupying different situations, in one case being located even above the level of the pons. They were, in fact, aberrant forms of infantile paralysis, and the point of interest was, firstly, that such varied forms of nerve disorder could arise from the same cause, showing that infantile paralysis was not so strictly a system disease as some had imagined, and, secondly, that infantile paralysis was a specific infective malady.

The President (Dr. Buzzard) said that the paper read by Dr. Pasteur was of much interest as being, he thought, the first occasion on which the infective character of the disease causing infantile paralysis had been brought before an English medical society. The cases related by Dr. Pasteur and those of Dr. Medin were mutually confirmatory of each other. He felt very sure that infantile paralysis arose from some toxic influence, and that this was not limited to the anterior cornua of the spinal cord. In Medin's cases 27 of them were typical of anterior poliomyelitis, showing that there could be no doubt as to the nature of the disease, yet in the rest of the cases the morbid changes were distributed over different portions of the cerebro-spinal system; and it seemed clear that some toxic agent, to which we could not yet give a name, was associated with a certain pyrexial disease, and might in some cases fall on the anterior cornua, while in others its effects might be distributed over different parts of the cerebral and other centres. He related a case in which a little boy was attacked with infantile paralysis and it was found that at the same time that he had the febrile stage a little sister had a febrile attack of the same nature, but not ending in paralysis. Dr. Buzzard said he was in the habit of directing his clerks to inquire not only as to antecedent illness among relatives, but also as to coincident illness among other children in the house or neighbourhood. Nothing could be more sad than the condition in which children were left for their lives by this disease, and it was of great importance that those who had the opportunity of seeing such cases at the very beginning should investigate the blood bacteriologically at the earliest possible moment, in the hope that some bacillary cause of the malady might be discovered, and that an anti-toxin might be found, by the immediate use of which some of these disastrous consequences might be prevented.

